# Outcome of segmentectomy vs. lobectomy in patients with cT1a-b N0 lung cancer and postoperative N1 upstaging

**DOI:** 10.1007/s00423-026-04089-0

**Published:** 2026-05-30

**Authors:** Hafsa Kaman, Theresa Stork, Özlem Okumus, Mehran Jemsi, Ana-Maria Petrone, Yazan Alnajdawi, Stéphane Collaud

**Affiliations:** Department of Thoracic Surgery, Cologne-Merheim Hospital, Lung Clinic, University of Witten/Herdecke, Ostmerheimer Str. 200, Cologne, 51109 Germany

**Keywords:** Lung cancer, Nodal upstaging, Segmentectomy, Lobectomy

## Abstract

**Purpose:**

Segmentectomy is offered in selected patients with clinical T1a-b N0 M0 lung cancer. In case of positive N1 lymph node at fresh frozen section, conversion to lobectomy is recommended. Indication for redo surgery and completion lobectomy in case of positive N1 lymph node at final pathology is highly debated. We aim to compare the outcome between segmentectomy and lobectomy in patients with cT1a–b N0 M0 lung cancer with postoperative N1-upstaging.

**Methods:**

We retrospectively reviewed all patients with pT1a-b N1cM0 lung cancer who underwent surgical resection with lymph node dissection between January 2013 and January 2024 at our institution. We included patients with tumor size ≤ 2 cm and postoperative N1-upstaging. Disease-free (DFS) and overall (OS) survivals were calculated from the date of surgery until recurrence or death and were compared between the groups.

**Results:**

Twenty-five patients with cT1a-b N0 M0 lung cancer and postoperative N1-upstaging were identified. Median age was 64 years (42–81). Sixteen (64%) patients underwent lobectomy There was no significant difference in tumor size (median: 1,1, IQR$\:[$1,1–1,8$\:]$ vs. 1,5, IQR$\:[$1,4 − 1,8$\:]$, *p* = 0.186) and FEV1% (median: 76, IQR$\:[$74- 92$\:]$ vs. 75, IQR$\:[$69- 87$\:]$, *p* = 0.656) between segmentectomy and lobectomy groups. Number of lymph nodes removed was significantly lower in the segmentectomy group (median: 10, IQR$\:[$4- 26$\:]$ vs. 23, IQR$\:[$14- 32$\:]$ *p* = 0.035). Median follow-up was 53, IQR$\:[$41- 63$\:]$ months. There was no difference in 5-year DFS (72.9%vs 70.5%, *p* = 0.502) and OS (85,7%vs. 85,6%, *p* = 0.233) between the groups.

**Conclusion:**

In patients who underwent segmentectomy or lobectomy for cT1a-b N0 M0 lung cancer and postoperative N1-upstaging, no difference in survivals was seen in our cohort.

## Introduction

Since 1995 lobectomy has been the treatment of choice for stage I non-small cell lung cancer, (NSCLC) according to the findings of The Lung Cancer Study Group [1]. This landmark study demonstrated that lobectomy provided superior local control and better survival outcomes compared with sublobar resections.

Recently, two major randomized trials: The JCOG0802/WJOG4607L [2] and CALGB140503 [3] trial, provided evidence demonstrating the noninferiority of sublobar resection compared to lobectomy in patients with early-stage NSCLC. Consequently, segmentectomy has been increasingly offered for patients with stage IA1–2 lung cancer, representing a substantial paradigm shift in the surgical management of early-stage lung cancer.

Despite the increasing adoption of segmentectomy for selected early-stage lung cancer, lobectomy is still the standard of care for patients with stage IIB lung cancer. Therefore, while performing a segmentectomy, intraoperative assessment of N1 lymph nodes is advised. When metastatic N1 disease is identified intraoperatively, proceeding with lobectomy rather than segmentectomy alone is recommended [4, 5]. Since postoperative lymph node upstaging can occur in up to 10% of patients with clinical stage I NSCLC [6], and as segmentectomy is increasingly performed for early stage lung cancer, the clinical scenario of postoperative nodal upstaging after segmentectomy is expected to become more frequent. How should patients be treated if they had segmentectomy and postoperative N1 upstaging? Is there an indication for completion lobectomy?

While completion lobectomy may improve local control by removing residual intralobar lymphatic pathway, and occult satellite lesions or remaining nodal tissue that may harbor microscopic disease, a redo operation to perform a completion lobectomy might jeopardize the fitness of patients to undergo adjuvant systemic therapy, which is a standard of care in stage IIB lung cancer with proven survival benefit [5].

A recent review of literature summarized contemporary studies comparing the outcomes after segmentectomy versus lobectomy for early-stage lung cancer showing largely comparable oncologic outcomes between segmentectomy and lobectomy in selected patients with occult nodal disease [7].

To contribute to the clinical decision process, we compared oncological outcomes after segmentectomy and lobectomy in patients subsequently upstaged to N1 following surgery in our institution.

## Materials and methods

We retrospectively reviewed all patients with pT1 pN1 cM0 NSCLC who underwent surgical resection with lymph node dissection between January 2013 and January 2024 at our institution. The study received approval from the ethic committee of the University of Witten Herdecke (approval number S-178/2024). Requirement for informed consent was waived due to the retrospective nature of the study.

Data were obtained from electronic patient records. All patients were restaged according to the 8th edition of the TNM classification [8]. Patients were excluded if their tumor size was > 2 cm and/or if preoperative clinical and/or pathological evidence of positive lymph node metastasis was present. We included patients who underwent segmentectomy or lobectomy. Patients after wedge resections were excluded.

Preoperative contrast enhanced chest computer tomography (CT) with upper abdomen was performed in all patients. Staging with positron emission tomography (PET/CT) or whole-body magnetic resonance imaging (MRI), cerebral CT or MRI as well as endobronchial ultrasonography (EBUS) were done, in case of central tumors or if lymph node involvement was suspected. Tumors were considered central if they were localized in the inner two thirds of the hemithorax according to the definition of The European Society of Thoracic Surgeons (ESTS) [9]. Consolidation to tumor ratio (CTR) was calculated on the initial chest CT-scan following the JCOG0201criteria, defined as the ratio of the maximum diameter of consolidation to the maximum diameter of the tumor [10].

Video-assisted or open segmentectomy and lobectomy with lymphadenectomy were performed at surgeon’s discretion. Intraoperative frozen section of lymph nodes was performed in case of suspected lymph node involvement. Lymphadenectomy was defined as lobe-specific systematic nodal dissection if at least six lymph nodes were retrieved, including hilar and intrapulmonary lymph nodes as well as the following mediastinal stations: stations 2R, 4R, and 7 for right upper and middle lobe tumors; stations 4R, 7, 8, and 9 for right lower lobe tumors; stations 5, 6, and 7 for left upper lobe tumors; and stations 7, 8, and 9 for left lower lobe tumors.

The following variables were collected: age, sex, smoking status, comorbidities using Charlson Deyo score, lung function, type of surgery, hospital stay, postoperative complications, clinical and pathological staging, number of dissected lymph nodes, station distribution of positive lymph node, presence of lymphovascular invasion, surgical margin.

Continuous variables were presented as means along with their standard deviation (SD) or as median with interquartile ranges (IQR) and were compared between groups using Student’s t-test and Mann-Whitney U test. Categorical variables were compared using chi-square test or Fisher’s exact test. Overall survival (OS) and disease-free survival (DFS) was calculated from the date of surgery and estimated using the Kaplan-Meier method. Log-rank was used to compare survivals. A p value of ≤0,05 was considered statistically significant. All statistical analyses were performed using IBM SPSS software (IBM Corp., Armonk, NY, USA). Graphical representations were generated using GraphPad Prism version 8.

## Results

We identified 505 patients with NSCLC with tumor size ≤ 2 cm who underwent a lobectomy or segmentectomy between January 2013 and January 2024 in our institution. Pathological N1-upstaging was observed in 25 (5%) patients, representing our study cohort. Of those, nine patients (36%) underwent segmentectomy and sixteen patients (64%) underwent lobectomy. Table 1 describes the distribution of patients and tumor parameters in both the lobectomy and segmentectomy groups.

**Table 1:** Patient and tumor characteristics for both lobectomy and segmentectomy groups

Covariate	Segmentectomy (*n* = 9)	Lobectomy (*n* = 16)	*P*-value
Age in years (mean+/- SD)	68. ($\:\pm\:9$)	62. ($\:\pm\:$9)	0.14
Gender n (%) Male	4 (56%)	9 (57%)	0.57
Smoking Status n (%) Never smoker Current smoker Former smoker	2 (22%) 1 (11%) 6 (67%)	1 (6%) 10 (63%) 5 (31%)	0.04
Charlson deyo score n (%) 0 1–2 3–4 >= 5	1 (11%) 0 5 (56%) 3 (33%)	2 (12%) 7 (44%) 6 (38%) 1 (6%)	0.17
FEV 1 (%) (median [IQR])	$\:76\:[74-92]$	$\:75\:[69-87]$	0.65
DLCO (%) (median [IQR])	$\:98\:[87-98]$	$\:73\:[63-81]$	0.005
Surgical approach VATS Thoracotomy Conversion	9 (100%) 0 0	14 (87%) 0 2 (13%)	0.26
Hospital length of stay in days (median [IQR])	7$\:\:[5-8]$	7$\:[6-9]$	0.95
ICU length of stay in days (median [IQR])	1$\:[0-1]$	1$\:[1-1]$	0.36
Complications n (%) No Yes	6 (86%) 1 (14%)	11 (85%) 2 (15%)	0.94
Tumour size cm(median [IQR])	$\:\mathrm{1,1}\:[\mathrm{1,1}-\mathrm{1,8}]$	$\:\mathrm{1,5}\:[\mathrm{1,4}-\mathrm{1,8}]$	0.18
Resection margin cm (median [IQR])	$\:\mathrm{0,4}\:[\mathrm{0,1}5-\mathrm{1,3}]$	$\:\mathrm{3,3}\:[\mathrm{0,8}-\mathrm{4,5}]$	0.29
Margin to tumour ratio (MRT) (n) MTR < 1 MRT ≥ 1	6 (67%) 3 (33%)	4 (25%) 12 (75%)	0.04
Resection margin status n (%) R0 RX	8 (89%) 1 (11%)	15 (94%) 1 (6%)	0.66
Number of resected lymph nodes (mean+/- SD)	14($\:\pm\:12$)	25 ($\:\pm\:12$)	0.03
Histology n (%) Adenocarcinoma Squamous cell carcinoma Other	5 (56%) 0 4 (44%)	9 (56%) 3 (19%) 4 (25%)	0.30
Adjuvant chemotherapy n (%) Received Not received	2 (25%) 6 (75%)	10 (67%) 5 (33%)	0.05

Abbreviations: *SD*= standard deviation, *IQR*= interquartile range, *FEV1* = forced expiratory volume in 1 s, *DLCO*= diffusion capacity of lungs for carbon monoxide, *VATS* = video assisted thoracoscopy

Surgery was performed mostly per VATS (92%). Conversion to open surgery took place in two patients in the lobectomy group for inadequate exposure.

All tumors in this cohort were solid (CTR = 1), and 72% were centrally located. While resection margin status did not differ significantly between lobectomy and segmentectomy groups (*p* = 0.66), achieving an adequate margin-to-tumor ratio (MTR ≥ 1) was significantly less frequent in the segmentectomy group compared to the lobectomy group: 3 (33%) vs. 12 (75%) patients, (*p* = 0.04).

The number of resected lymph nodes was significantly lower in the segmentectomy group (*p* = 0.03). Only two patients did not meet the definition for lobe specific systemic lymph node dissection due to retrieval of fewer than six lymph nodes, both patients belonged to the segmentectomy group. No significant difference was observed in the distribution of the affected stations (station 10 vs. > = 11) (*p* = 0,509).

In the segmentectomy group, two patients (25%) received adjuvant chemotherapy, compared to 11 patients (67%) in the lobectomy group (*p* = 0.05). Among segmentectomy patients who did not receive adjuvant therapy, two had typical carcinoid tumors, one was undergoing treatment for concurrent tuberculosis, and in four cases the reason was unknown. In the lobectomy group, five patients did not receive chemotherapy: one had a typical carcinoid, two refused, one had too many relevant comorbidities, and in one case the reason was unknown.

Recurrence occurred in 3 patients (33%) in the segmentectomy group and 3 patients (19%) in the lobectomy group (*p* = 0.41). Local recurrence was observed in one patient in the segmentectomy group (11%) and one patient in the lobectomy group (6%) (*p* = 0.71).

**Fig. 1:**
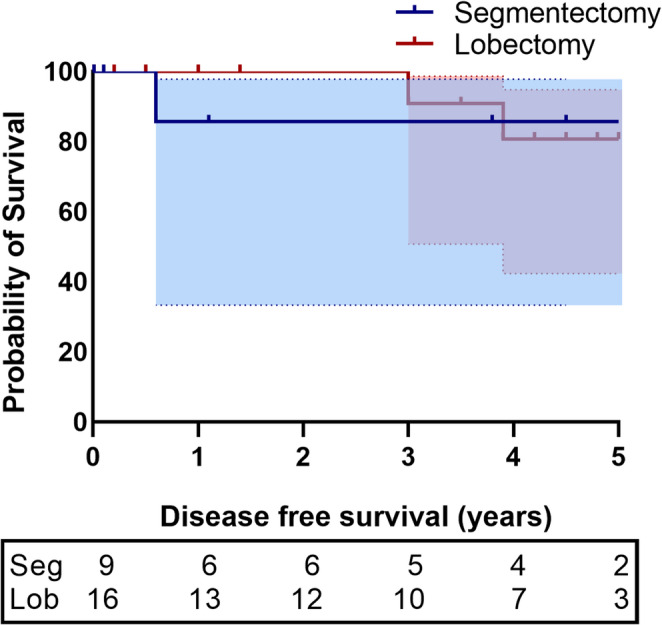
Kaplan-Meier estimate of disease-free survival Seg = Segmentectomy; Lob=Lobectomy

Ninety-days mortality was 0% in both groups. The median follow-up was 53 IQR $\:[$41–63$\:]$ months. Figure 1 shows the Kaplan-Meier estimate of DFS in segmentectomy and lobectomy groups.

 DFS was not significantly different between patients who underwent segmentectomy compared to lobectomy (73% vs. 70% *p* = 0,50).

**Fig. 2:**
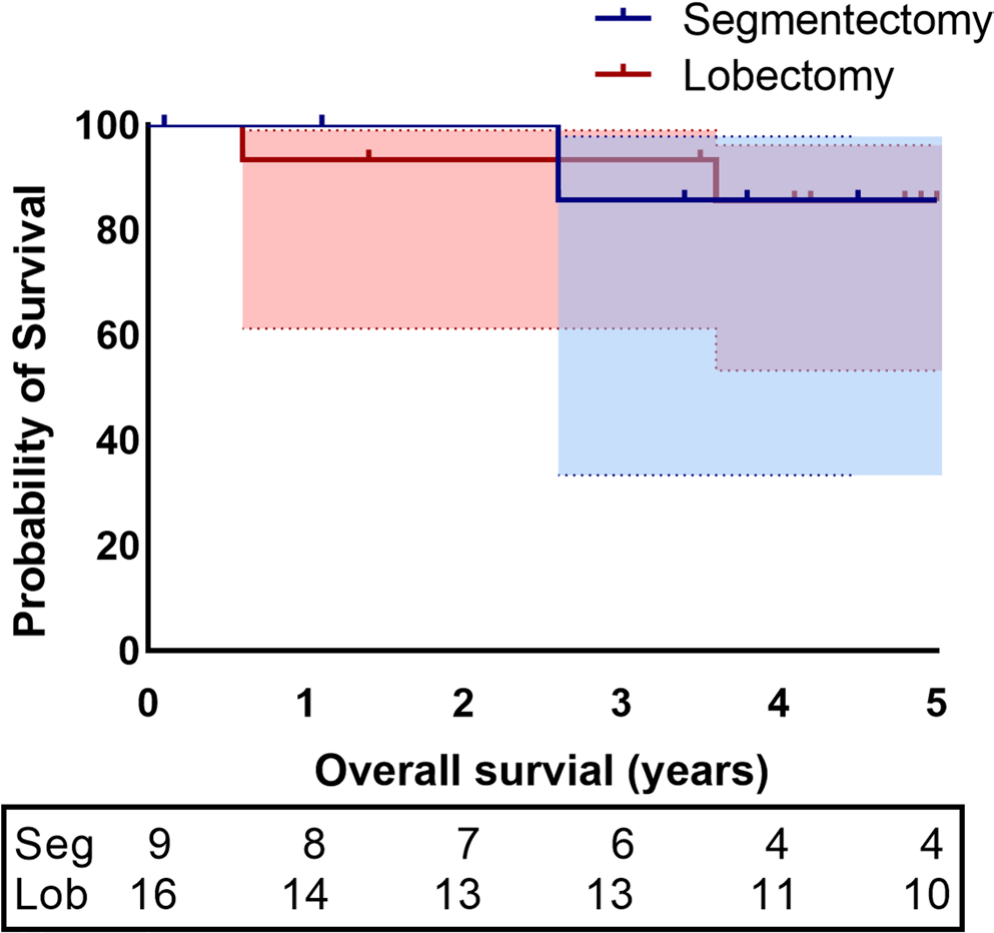
Kaplan-Meier estimate of overall survival, Seg =Segmentectomy; Lob=Lobectomy

Figure 2 shows the Kaplan-Meier estimate of OS in segmentectomy and lobectomy groups.

 There was no statistically significant difference in OS between both groups (86 vs 86% p=0,23).

## Discussion

 With the expansion of lung cancer screening programs and increase in diagnosis of early-stage lung cancer, segmentectomy is increasingly performed [11]. Experts recommend performing a frozen section of lymph nodes at the foot of the segmental bronchus to exclude N1 disease in intended segmentectomy. If the frozen section is positive, lobectomy should be performed instead of segmentectomy to reduce the risk of local recurrence [4]. However, although frozen section has a high diagnostic yield, its sensitivity remains low, with 80% [12, 13] leaving room for postoperative upstaging. Most importantly, frozen section is not performed routinely in most institutions due to logistical factors. As a result, postoperative nodal upstaging after segmentectomy remains a clinically relevant issue. Whether it is necessary to perform a completion lobectomy in case of pathologic nodal upstaging is still under debate [4]. Here, we compared the oncological outcomes of segmentectomy and lobectomy in patients with cT1a-b N0 M0 who had postoperative N1-upstaging. To ensure a homogeneous cohort, we focused exclusively on N1-upstaging, whereas prior studies often included both N2 nodal and T upstaging [14–17]. 

 The incidence of N1-nodal upstaging in our cohort was 5%. This aligns with findings in other studies. The Dutch Lung Surgery Audit reported a 6,2% rate of N1-nodal upstaging in tumors ≤ 2 cm [18]. The rate reported in the JCOG trial was 5%[19]. In a study to establish a clinical nomogram for predicting nodal upstaging in patients with NSCLC ≤ 2 cm, Ai et al. found a rate of 3,4% [12]. Robinson et al. investigated the prevalence of N1 nodal upstaging in peripheral clinical N0 NSCLC ≤ 2 cm, identifying patients prospectively through preregistration eligibility screening for the Alliance/CALGB 140503 trial, and reported an incidence of 14% [20]. This emphasizes the clinical relevance of the issue.

 In our retrospective study we did not observe a statistically significant difference in 5 years DFS or OS between segmentectomy and lobectomy groups. However, these findings must be interpreted cautiously given the retrospective design and the limited sample size. Larger retrospective studies have been conducted based on the national cancer database (NCDB). They included patients in clinical stage IA NSCLC and lacked data on recurrence, since only data on overall survival has been collected. This data suggests that there was no significant difference in overall survival between lobectomy and segmentectomy in clinical stage IA with postoperative nodal upstaging [7, 15–17]. 

 The rationale behind completion lobectomy is to achieve local control by eliminating potential remaining lymphatic tissue that could contain microscopic metastatic disease and prevent residual intrapulmonary lymphatic spread. However, Nomori et al. compared 10 patients who underwent completion lobectomy with 5 patients treated with segmentectomy despite nodal upstaging [14] . Among patients with completion lobectomy, no additional lymph node metastases were identified in the specimen. In the segmentectomy group, two patients experienced distant recurrence, while no local recurrence was observed, suggesting that segmentectomy provided comparable local control to lobectomy in this cohort. Consistent with these findings, in our study the rate of local recurrences was not statistically significant between the segmentectomy and lobectomy groups (p= 0.71) although achieving a margin-to-tumor ratio ≥1 was less frequent in the segmentectomy group (p=0.04). This observation might be explained by the small tumor size (≤2 cm) or more likely due to the small sample size. 

 Our findings and those of previous authors emphasize the importance of lymph node dissection when performing segmentectomies, to avoid overseeing nodal disease. In our series, although the number of examined lymph nodes was significantly higher in the lobectomy group, the distribution of the examined stations was similar. In fact, while performing a segmentectomy, adequate lymph node dissection is encouraged to diagnose nodal upstaging, thus permitting the patient to access adjuvant therapy, which seems to be a more decisive factor of the outcome as the extent of resection itself. Indeed, Razi et al conducted a study to compare the outcome of lobectomy and segmentectomy for clinical T1N0M0 NSCLC with postoperative nodal upstaging. In multivariable analysis adjuvant chemotherapy was associated with better survival for patients with unsuspected N1 (hazard ratio, 0.613; 95%confidence interval, 0.536-0.700; P<.001), while the extent of resection did not impact survival (p=0.71) [17]. The presence of nodal disease is proof of systemic spread, which probably takes a toll on the prognosis and might explain the similarity of survival between the groups regardless of the extent of resection. In our cohort, this effect could not be reproduced, likely due to the limited sample size. 

 While completion lobectomy can be technically challenging, this procedure is safe in expert hands [21–23]. However, reoperations on patients with pre-existing comorbidities can raise the perioperative morbidity and mortality [24], which might jeopardize the suitability of these patients to postoperative therapy. Knowing that adjuvant therapy offers a clear survival benefit in stage IIB NSCLC [25], performing a completion lobectomy must be questioned based on results of the literature and ours. 

 Our study has some limitations due to its retrospective nature and small sample size, which limit statistical power. Some baseline differences between the groups such as the pulmonary function and smoking status reflect inherent selection bias related to the non-randomized retrospective design of the study. The study spans a 10-year period, during which treatment practices particularly the use of adjuvant systemic therapy have undergone significant evolution. Additionally, we did include a subset of patients (n=3, two in the segmentectomy group and one in the lobectomy group) with typical carcinoid tumors, which are characterized by more favorable outcomes compared to conventional NSCLC and may have biased our results. Detailed information regarding the specific indications for segmentectomy before 2022 and the reasons why some patients did not receive adjuvant therapy were not consistently available, which limits the interpretation of the result. Given these constraints, the observed similarities in DFS and OS should not be interpreted as evidence of equivalence between segmentectomy and lobectomy. These results should be considered as exploratory and hypothesis generating. 

## Conclusion

In patients with cT1a-bN0M0 and pathologic N1 nodal upstaging, there was no statistically significant difference in DFS and OS in patients undergoing segmentectomy or lobectomy. Since large prospective studies will likely not be feasible, the decision to proceed or not to completion lobectomy after postoperative N1 upstaging should take place within multidisciplinary tumor board and be highly individualized taking tumor biology and patient characteristics into account. 

## Data Availability

No datasets were generated or analysed during the current study.
